# How to Measure the Psychological “Flow”? A Neuroscience Perspective

**DOI:** 10.3389/fpsyg.2016.01823

**Published:** 2016-12-06

**Authors:** Guy Cheron

**Affiliations:** ^1^Laboratory of Neurophysiology and Movement Biomechanics, ULB Neuroscience Institute, Université Libre de BruxellesBrussels, Belgium; ^2^Laboratory of Electrophysiology, Université de Mons-HainautMons, Belgium

**Keywords:** flow, EEG, EMG, synchrony, stress

## Introduction

The term “flow” as conceptualized by Csikszentmihalyi (1975) describes the optimal experiences that are most enjoyable in human life while fully engaging in an activity (Csikszentmihalyi, 1978, 1990, 1998). Within athletic, artistic and occupational behaviors, the flow emerges from an action that requires specific skills and challenges (Marin and Bhattacharya, 2013). It also expands self-esteem and the individual's capabilities through learning new optimizations that increase the feelings of continuity and fluidity in attention and action. The flow does not occur for all types of behavior. It requires clear goals, unambiguous and immediate feedback, assuming a perfect match between skills and challenge (Mao et al., 2016).

In contrast to its behavioral counterpart, commonly expressed by the term “stress,” the flow may be viewed as a convergent physiological entity supported by the emergence of a unique brain state. Since flow requires challenges, it must be supported by short-term stress (the good one) that assumes physiological protection (e.g., enhancement of immunoprotection) to deal with challenges. On the contrary, long-term stress (chronic) impinges on reaching the flow state and disrupts the immunoprotective effects on various physiological functions (Dhabhar, 2014). Because of the conjunction of action skill, challenge and emotion in a single flow-state, the scientific community remains confronted with the complex question of identifying its neurophysiological outcomes. This challenge is in line with the unresolved questions relating neurometric-psychometric comparisons in an attempt to identify neurophysiological activities and sensations (Stüttgen et al., 2011) that occur during the flow.

In this Grand Challenges monograph, my intent is to trace experimental perspectives applying tools of movement neuroscience (Cheron, 2015; Cheron et al., 2016) in order to characterize the physiological aspects of the brain state during flow in sports.

## The electromyographic signals (EMG) as a prediction of flow perception

To move our body in everyday situations, a functional tradeoff between external (e.g., gravity) and internal force (e.g., muscular torques) must be continuously controlled. The perception of flow would emerge in a particular physiological state where (1) the ascending somesthetic signals including graviception, (2) the descending motor commands, and (3) an appropriate central resting state, including memorized items, would combine to reach the flow consciousness. As the flow sensation goes along with or follows movement, the initial intention must be translated to the muscles in order to generate forces and displacements. The subsequent environmental changes produce feedback sensations which close the loop between action and sensation (Schwartz, 2016).

Among these three complex signals, the surface EMG recording of multiple muscles may help to quantify the final output signals coming from different motoneuron pools. These signals not only represent the descending motor commands, but also the integration of the re-afferent signals coming from the peripheral sensors (Chéron and Godaux, 1986a). For some authors, the EMG signals represent pre-programmed commands used by the CNS for controlling movement (Chéron and Godaux, 1986b; Gottlieb, 1998a,b; Pfann et al., 1998; Cheron et al., 2007), while for those supporting the equilibrium point hypothesis (Feldman, 1986; Feldman et al., 2013; Ambike et al., 2016) it represents an emergent property of the system, and not the controlled variable of the movement. Regardless of this unresolved debate, the close relationship between EMG signals and the primary motor cortex (M1) has recently been supported by simultaneous recording of the corticomotoneuronal (CM) cells of M1 and their monosynaptic targeted motoneurons in alert monkeys (Griffin et al., 2015). These authors demonstrated that some CM cells were selectively activated when the targeted muscle was used as an agonist, while other CM cells when the same muscle were used as an antagonist, fixator or synergist. Positive or negative synchronization of M1 cell pairs, assume the existence of synchrony in the motor cortex related to muscle action (Jackson et al., 2003), facilitating the recording of EEG oscillations from the motor cortex in relation to the EMG pattern. In this context, a recent study of Moscatelli et al. (2016) demonstrated greater corticospinal excitability in karate athletes with respect to controls, indicating a sport-specific adaptation between inhibitory and excitatory network in modulating the final command from M1. When comparing professional handball players and ballet dancers, Meier et al. (2016) demonstrated neuroplastic adaptations in the gray matter (GM) representation and corticospinal path (CP) of the foot and the hand area depending on sport practice. GM volume and CP density were respectively more important in hand areas of handball experts and in foot areas of ballet dancers. This sport-specific dependency of the corticospinal commands (Hänggi et al., 2010, 2015; Bar and DeSouza, 2016; Meier et al., 2016) should be taken into account in the flow state research.

The sculpting of the EMG signals into the classical triphasic EMG burst on antagonistic muscles producing rapid and self-terminated movements may serve as an experimental prototype for the study of the flow sensation emergence. Indeed, in the context of a single joint movement directed to a final position respecting the “as fast as possible” consign, the duration and the amplitude of the three main bursts, (action pulse, PA of the agonist muscle; the braking pulse, PB of the antagonist muscle and the clamping pulse, PC of the agonist muscle) may characterize the final performance in terms of velocity or precision (Hannaford et al., 1985; Chéron and Godaux, 1986b).

The training of these rapid movements induced reduction of movement time, movement error, variability in amplitude, and was accompanied by reduction of PA and PC duration and PB and PC latencies (Liang et al., 2008). These modifications are expected to be associated with the flow sensation. Such triphasic EMG bursts have been successfully simulated with a dynamic recurrent neural network (DRNN) (Cheron et al., 2007). In this context, the different movement trials associated with psychological assessment of subjects' feelings about their performance can be introduced into the DRNN in order to determine the proper dynamics related to the best score or the best self-esteem.

Recently, Toma and Lacquaniti (2016) have used the sense of muscular effort as psychophysical performance in order to correlate EMG signals and perceptual decisions. Indeed, EMG signals are also representative of the functioning of the primary motor cortex (M1) and relate to force production and the sense of effort as previously demonstrated by the application of repetitive transcranial magnetic stimulation (rTMS) (Nowak et al., 2005; Goodall et al., 2013). In particular, rTMS applied at slow frequency on M1 produced an overestimation of the exerted force (Takarada et al., 2014). In contrast, theta-burst TMS applied on the supplementary motor area (SMA) induced a decrease in the sense of effort (Zénon et al., 2015) and an increase in vibration sensation. The same type of TMS also reduced the amplitude of the somatosensory N30 component (Legon et al., 2013) partly originated in SMA (Kanovský et al., 2003; Cebolla et al., 2011) and considered as a dopaminergic biomarker (Cheron, 1999; Pierantozzi et al., 1999). These TMS experiments and the responses of related evoked potentials strongly indicated that M1 and SMA are good candidates for producing the sense of flow (see below).

The new method proposed by Toma and Lacquaniti, (2016) allows researchers to quantify a “muscle-metric function” related to psychological decisions. It would be thus possible to capture trial-by-trial EMG modulation associated with trial-by-trial psychological judgments for which the different questionnaires about the flow perception would be applied (e.g., the Jackson's Flow State Scale (FSS) (Jackson, 1996; Tenenbaum et al., 1999).

## The internal model concept and the flow

Another advantage of EMG recording is that it correlates well with the formation of the internal model (IM). The IM is a central concept in motor control (Wolpert et al., 1995, 1998; Ishikawa et al., 2016) developed in different experimental fields. It may help to circumscribe the emergency of flow because it contributes to building new dynamic attitudes based on past experiments, it may also allow some motor creativity to go “in the zone.”

Acting with a manipulandum inside of a controlled force field is an appropriate situation to study the relationships between EMG patterns and the adaptation of the IM. It was demonstrated that the IM formation was first accompanied by EMG patterns counteracting the force field initially driven by feedback error signal (Thoroughman and Shadmehr, 1999) before being shifted in advance of the movement in response of a feedforward command. This predictive function of the IM reduces the co-activation and spatially tunes the EMG of each muscle in function of the direction of learned force field. We may reasonably postulate that the flow sensation occurs when the IM output perfectly fits the re-afferent input. As IMs are dynamics entities, they use flexible combinations of motor primitives resulting from the transformation of the desired movement trajectories into motor commands (Thoroughman and Shadmehr, 2000; Degallier and Ijspeert, 2010; Hogan and Sternad, 2012).

For reaching the flow, the CNS must generate fluent and skilled motor commands for which different challenges regarding the sensorimotor control need to be encompassed. These problems include the presence of noise, uncertainty, redundancy, nonlinearity, nonstationarity and delay in neuronal loops (Franklin and Wolpert, 2011). These authors demonstrated that fluent and efficient sensorimotor behavior can be reached by three types of control (feedback, impedance, predictive), referred to the sensorimotor learning and the Bayesian Decision theory.

As it was demonstrated that subjects internally use the statistical distribution of the information related to the task and their sensorimotor uncertainty in order to realize a Bayesian optimization process during learning (Körding and Wolpert, 2004; Orbán and Wolpert, 2011), we may suggest that the flow occurs in some exceptional circumstances on top of this process.

The self-experiencing of the flow provides a vivid impression that this sensation occurs simultaneously or in line with the accomplishment of the movement and independent of the completion of the entire action. For example, during the realization of a “jibe,” a classical skilled movement in windsurfing, the flow may emerge when the turning trajectory is accomplished at high speed, but with a smoothness profile encompassing total involvement of the whole body. The flow is thus experienced before the final success or failure occurring at the end of the action.

Smoothness perception seems to be on the root of the flow sensation. This perception happens when the movement is performed continuously without any interruption (Balasubramanian et al., 2012, 2015). The recording of movement kinematics may then well characterize the bell-shaped speed profile of smooth movement. The smoothness can be robustly quantified by Fourier speed profile spectrum (Balasubramanian et al., 2012). In spite of the fact that some movement tasks required inherent intermittencies marked by a dip in the speed profile, inside of a specific task these movement intermittencies could be indicative of the difficulty for producing smoothness compatible with the flow.

Peripheral and central fatigue must also be taken into account. If movement repetition may facilitate the flow emergence, it also introduces, fatigue elements compromising the entry into “the zone.” The studies of Amann et al. (2006, 2007) and the related viewpoint defended by Noakes and Marino (2007) illustrates the complexity of the interaction between multiple physiological factors of peripheral and central fatigue. In this context, Torres-Peralta et al. (2016) recently demonstrated that EMG alterations during isokinetic sprint were not due to lactate accumulation or muscle acidification but to central fatigue (see also Morales-Alamo et al., 2015).

## How to catch the flow with EEG dynamics

The initial definition of the psychological flow imposes to assess whether EEG rhythms are simultaneously correlates with perception and action into a single behavioral task. Two main and complementary EEG approaches, both supported by EEGLab software (Delorme and Makeig, 2004), consist of the quantification of the (1) power and (2) the phase of the different frequency EEG oscillations ranging from delta, theta, beta and gamma bands occurring before, during and after the flow. The power spectral modulations are expressed by increase (event-related synchronization, ERS) or decrease (event-related desynchronization, ERD) (Klimesch et al., 1990, 1997; Pfurtscheller and Neuper, 1994; Neuper et al., 2006) measured with respect to a “neutral” baseline occurring before a behavioral event or by comparing the power modulation related to the same event, but appearing during two different brain states (Cebolla et al., 2011, 2014). These experimental approaches should be applied in the behavioral context of the flow and will offer the opportunity to decipher the neurophysiological mechanisms at the basis of flow emergence. For example, when a power modulation is recorded in a specific EEG frequency band in response to a behavioral event, but without any concomitant phase-locking of the same oscillation, it is possible to infer that the modulation is well related to the event but that no prediction or top-down are implicated. On contrary, when a phase-locking (measured by the inter-trials coherency, ITC) is present in a specific frequency band without any concomitant power modulation of the same oscillation (pure phase-locking) (Cebolla et al., 2009). It is possible to assume that the ongoing EEG oscillation is implicated by a predictive shift of the phase indicating the presence of a top-down mechanism (Bonnefond and Jensen, 2015). As a specific oscillation cannot be phase-locked to two different types of events occurring in the same period of time (see beta-gamma oscillation during movement gating (Cebolla et al., 2009), it should also be possible to decipher which type of oscillation is implicated in the flow emergence. Based on the fact that the CNS uses a multiplexing of different oscillations (Wimmer et al., 2016) and that the phase-locking of slower ongoing oscillations modulates the power of faster oscillations, it is highly conceivable that the EEG “flow signature” will be polyrhythmic and supported by different mechanisms. The role played by theta oscillations modulated by dopamine in the functional synchronization of the hippocampus and the frontal cortex (Benchenane et al., 2011; Fujisawa and Buzsáki, 2011) must be taken into account in the future search of the “flow signature.” In relation to the importance of dopaminergic influence in positive thinking, it is interesting to note that alpha and beta EEG rhythms recorded in the centro-parietal scalp regions of Parkinsonian patient were increased after L-dopa administration (Melgari et al., 2014).

Among the different EEG rhythms recently revisited in sport performance (Cheron et al., 2016), beta oscillation (13–30 Hz) is probably another relevant candidate for indexing the flow. The ERS/ERD modulation in this frequency range was increased after training (Moisello et al., 2015), a condition that must facilitate the flow emergence.

## Corticomuscular coherence and flow identification

Another interesting approach using information from both EMG and EEG signals is based on corticomuscular coherence (CMC), i.e., frequency domain estimation of the functional coupling between EEG oscillations and the active muscles EMG (Mima and Hallett, 1999). More specifically, CMC in the beta oscillation range (13–30 Hz) is recognized as a communicational index between cortical motor areas and muscles (Onushko et al., 2013; Jacobs et al., 2015) CMC in the beta (15–30 Hz) range between motor cortex and hand muscles during precision grip task was positively increased with the lever of compliance (Kilner et al., 2000). It was recently reported that the practice performance of the manual dexterity promoted by smartphone and tablet operations increases the CMC between beta EEG and hand muscles (Larsen et al., 2016).

CMC has also been reported in the gamma range (35–60 Hz) (magnetoencephalographic (MEG) signals) recorded in the contralateral motor cortex and the 35–60 Hz EMG of forearm muscles during maximal isometric contractions (Brown et al., 1998; Brown, 2000; Brown and Marsden, 2001). As the performance of a skilled movement implies the synchronization of neural oscillations spatially distributed in different sites (Brown and Marsden, 2001), the emergence of the flow could be accompanied by highly specific cortico-cortical long-range synchrony (Harris and Gordon, 2015) or coherency. Different methodologies (Nolte et al., 2004; Stam et al., 2007; Ewald et al., 2012) are now able to approach the temporal evolution of the cortico-cortical coherency forming a functional network in which the direction of the communication flux can be established (Cheron et al., 2014; Hillebrand et al., 2016).

## Neurofeedback and flow training

Three recent reviews of Gruzelier (2014a,b,c) have paved the way for the application of appropriate EEG neurofeedback not only in BCI or rehabilitation domains but also in the search for different mental attitudes that are required for flow emergence, such as improvement of emotional-commitment, stage presence, expressiveness and creativity in music performance (Gruzelier et al., 2014). The same group (Gruzelier et al., 2010) demonstrated that neurofeedback training of the sensorimotor rhythm (SMR, 12–15 Hz) in virtual reality increased the Flow State scale score on the self-ratings sense of control. SMR training consists of providing feedback indication of three different frequency band powers at the same time (beta, 22–30 Hz; SMR, 12–15 Hz and theta, 4–7 Hz) and rewarding subjects only if the SMR is enhanced without any concurrent increase of theta and beta oscillations (Gruzelier et al., 2014).

In summary, the characterization of the flow state will benefit from the development of a new dialogue between psychometric and neurometric approaches. This dialogue should be promoted by new experimental paradigms taking account of the specificity of athletic, artistic or occupational behaviors during flow emergence. The recording of multiple biological signals from the brain and the muscle during movement measured in a well-defined environment should better define the neurophysiological signature of the flow and facilitate training procedures for reaching this optimal sensation. Therefore, the role to be played by this Specialty Grand Challenge in Frontiers in Movement Neuroscience and Sport Psychology is to provide an ideal platform for sharing the scientific outcomes in the search of the flow. It will also promote clear and high impact publications including new methods, experimental paradigms and scientific debates about this unique mental state encountered in different behavioral contexts. These future publications will also facilitate the training procedures for reaching this optimal sensation.

## Author contributions

The author confirms being the sole contributor of this work and approved it for publication.

## Funding

I would like to thank A. Leroy and B. Dan for their fruitful discussion about the manuscript, T. D'Angelo, M. Dufief, E. Toussaint, E. Hortmanns, and M. Petieau, for their expert technical assistance. This work was funded by the Belgian Federal Science Policy Office, the European Space Agency (AO-2004, 118), the Belgian National Fund for Scientific Research (FNRS), the research funds of the Université Libre de Bruxelles and of the Université de Mons (Belgium), the FEDER support (BIOFACT), the MINDWALKER project (FP7–2007–2013) supported by the European Commission, the Fonds G. Leibu and the NeuroAtt BIOWIN project supported by the Walloon Country.

### Conflict of interest statement

The author declares that the research was conducted in the absence of any commercial or financial relationships that could be construed as a potential conflict of interest. The handling Editor declared a shared affiliation, though no other collaboration, with the author GC and states that the process nevertheless met the standards of a fair and objective review.
